# Oat Brewery Waste Decreased Methane Production and Alters Rumen Fermentation, Microbiota Composition, and CAZymes Profiles

**DOI:** 10.3390/microorganisms12071475

**Published:** 2024-07-19

**Authors:** Pradeep Kumar Malik, Shraddha Trivedi, Archit Mohapatra, Atul Purshottam Kolte, Anjumoni Mech, Tsuma Victor, Elena Ahasic, Raghavendra Bhatta

**Affiliations:** 1 ICAR-National Institute of Animal Nutrition and Physiology, Bangalore 560030, India; shraddha.trivedi_8@yahoo.com (S.T.); arch10moha@gmail.com (A.M.); atulkolte@gmail.com (A.P.K.); anjumoni_0071@rediffmail.com (A.M.); 2 International Atomic Energy Agency, Vienna International Centre, P.O. Box 100, A-1400 Vienna, Austria; v.tsuma@iaea.org (T.V.); e.ahasic@iaea.org (E.A.); 3 Indian Council of Agricultural Research, New Delhi 110001, India; ragha0209@yahoo.com

**Keywords:** bioresource use, feed, oat brewery waste, methane, microbiota

## Abstract

The transformation of oat brewery waste (OBW) into livestock feed could be a potential replacement for the expensive concentrate and one of the effective approaches for avoiding health hazards due to the accumulation of oat brewery waste in the environment. To explore the potential of OBW as a methane (CH_4_) mitigating agent, an in vitro study was undertaken to investigate the effect of graded replacement of concentrate with OBW on CH_4_ production, microbiota, feed fermentation, and CAZymes. A total of five treatments with variable proportions of OBW were formulated. The results indicated a linear decrease in the total gas production and a 38–52% decrease in CH_4_ production with a 60 and 100% replacement of concentrate with OBW. The inclusion of OBW also affected the abundance of microbes such as Firmicutes, Euryarchaeota, *Methanobrevibacter*, and protozoa numbers. This study demonstrated that OBW can partially replace the concentrate and effectively mitigate CH_4_ production; however, the concurrent decrease in fermentation cautioned for the partial replacement of concentrate with OBW at an appropriate level at which the fermentation remains unaffected while decreasing CH_4_ production. Therefore, waste from oat breweries can contribute to curtailing the accumulation of greenhouse gases (GHGs) in the atmosphere.

## 1. Introduction

India possesses the largest livestock population in the world [1], majorly reared by small and marginal farmers who either have no land or extremely small holdings (<2 hectares) or cultivate the fodder for their livestock. The country is facing an acute shortage of concentrate, dry, and green fodder [2], which is further aggravated by the food-feed competition [3]. Indian livestock primarily thrives on crop residue-based diets [4], which are not only deficient in critical nutrients but also produce very high emissions of enteric CH_4_. 

With an average concentration of 1.89 ppm [5], CH_4_ is the second most prevalent greenhouse gas in the atmosphere [6,7]. The atmospheric concentration of CH_4_ is steadily rising at a rate of 10–13 ppb per year [8]. However, during 2020, an increase of more than 15 ppb was recorded [9]. Livestock contributes to approximately one-third of the CH_4_ emissions due to human activities [10]. In agriculture, enteric fermentation with an average emission of ~90 Tg, remains the single largest source of CH_4_ emissions [11]. Each liter of CH_4_ production takes 55 megajoules of energy away from the host animal [12].

The concentrate mixture, due to food-feed-fuel competition, is very expensive, and the marginal farmers cannot afford to feed the livestock on a concentrate-based diet. Waste from the brewing industry may be a potential feed resource and agent for CH_4_ mitigation. The brewing sector annually produces 40 million metric tons of brewery waste [13,14]. The transformation of brewery waste into livestock feed can help mitigate the health hazards arising from the accumulation of brewery biowaste in the environment. The feeding cost, however, shall be minimized using brewery waste that has a higher nutritional content than the foundation grain [15]. In addition to its high nutritional value, brewery waste due to high phenolic content [16] may affect the rumen microbiota and methanogenesis. 

Oat is a typical cereal crop that grows in a relatively damp climate and has a lower food value than barley [17]. Oat fodder is used to feed livestock, whereas grain can be used for both human and animal consumption. During beer production, oat grains, after the initial process of soaking, germination, and cooking, are crushed to extract the sugars and proteins. This process of beer-making generates huge waste in the brewery industry. Oat brewery waste (OBW) contains a high amount of protein and fiber and could be used as animal feed. The high protein content in OBW may fulfill a significant portion of the protein requirement and can also provide the fiber required for the bulk of the ruminant’s diet. To the best of our knowledge, the potential of OBW as a substitute for the expensive concentrate mixture and CH_4_ mitigating agent has never been explored, and this is probably the maiden attempt to report the impact of OBW on the rumen microbes, CH_4_ production, and other associated parameters. Therefore, the present study was designed to investigate the effect of graded replacement of conventional concentrate mixture with OBW on the rumen microbiota, CH_4_ production, fermentation, and CAZymes profiles. 

## 2. Materials and Methods

### 2.1. Test Material, Treatments and Composition

The OBW was procured from the brewery Industry located in Bangalore, India, and brought to the laboratory for evaluation. The initial moisture content was determined by drying in a hot air oven at 70 °C overnight, and the moisture content was expressed as a percentage. Simultaneously, another set of OBW was dried in the open air to monitor the moisture loss and ascertain if the fungal infestation had developed. To conduct in vitro studies, a concentrated mixture was formulated using maize grain (320 g/kg), soybean meal (130 g/kg), groundnut cake (120 g/kg), wheat bran (400 g/kg), mineral mixture (20 g/kg) and salt (10 g/kg). An initial in vitro study was carried out to compare the total gas and CH_4_ production among the shed and oven-dried OBW and concentrate mixture. Based on the preliminary results, the shed-dried OBW was used for the subsequent in vitro studies. A total of five treatments were formulated with the variable proportions (%) of concentrate and OBW: C (100 concentrate—0 OBW), T_1_ (80 concentrate—20 OBW), T_2_ (60 concentrate—40 OBW), T_3_ (40 concentrate—60 OBW), T_4_ (0 concentrate –100 OBW).

The chemical composition of OBW, concentrate, and treatments with variable proportions of concentrate and OBW (T_1_–T_4_) was analyzed in triplicate following the standard procedures. The ash content was determined according to AOAC [18], and the organic matter was estimated by the difference in the initial and final weight. The nitrogen content was determined as per AOAC [19] using an automatic nitrogen analyzer (IR digestion unit, Vapodest 450 & Titroline 5000, C. Gerhardt GmbH & Co. KG, Königswinter, Germany), and the CP was estimated by multiplying the nitrogen content with 6.25. The crude fiber (CF) and fiber fractions such as neutral detergent fiber (NDF) and acid detergent fiber (ADF) were determined according to AOAC [19] Van Soest et al. [20], respectively by an automatic fiber analyzer (Fibretherm FT12, C. Gerhardt GmbH & Co. KG, Königswinter, Germany).

### 2.2. Rumen Fluid Donor

Rumen fluid consisting of both solid and liquid fractions was collected 3 h post-feeding from two cannulated Holstein Friesian male adult cattle (BW ± SD 613 ± 15 kg) on the day of setting up in vitro incubations. The donor animals were fed on a mixture of Para (*Brachiaria mutica*) grass and hybrid Napier (*Pennisetum purpureum*) grass and a concentrated mixture in 70:30 (DM basis) to fulfill the maintenance requirement as per ICAR [21]. The para and hybrid Napier grass were mixed in equal proportion on a DM basis and finally constituted 70% of the diet. The composition of the concentrate mixture was the same as stated in the previous section. The feed was offered in the morning at 08.00 h, and the animals had free access to clean drinking water throughout the day. The rumen fluid was collected into a pre-warmed (39 °C) thermos flask purged with CO_2_ to maintain the anaerobic condition and brought to the laboratory. The rumen fluid containing the solid and liquid fractions in a 1:2 ratio was filtered through a double layer of muslin cloth, and the filtrate was collected in a glass vessel placed on a heating plate at 39 °C with a continuous flow of CO_2_. The rumen fluid served as a source of microbial inoculum for the in vitro studies. The buffer, macro, and micro mineral solutions were prepared on the previous day of incubation in accordance with Menke et al. [22] and stored at 39 °C. The buffer solution was prepared by weighing, mixing, and dissolving NaHCO_3_ (35.0 g) and NH_4_HCO_3_ (4.0 g) to make up the final volume of one liter with distilled water. The macro mineral solution was prepared by taking Na_2_HPO_4_ (5.7 g), KH_2_PO_4_ (6.2 g)7H_2_O (0.6 g) and dissolved into distilled water to make the final volume of one litre. Similarly, the micro mineral solution was prepared by using CaCl_2_.2H_2_O (13.2 g), MnCl_2_.4H_2_O (10.0 g), COCl_2_.6H_2_O (1.0 g), and FeCl_3_.6H_2_O (8.0 g) and made up the final volume 100 mL with distilled water. 

### 2.3. Total Gas and CH_4_ Production

About 200 mg dry samples, as outlined above, were weighed (Denver Instrument GmbH, Gottingen, Germany) and individually placed in a 100 mL glass syringe (Haberle, RSA cutting technologies GmbH, Schwerte, Germany). For each treatment (C, T_1_–T_4_), a total of six replicates were prepared for the incubation. Simultaneously, six syringes of blank contained buffered rumen inoculum without feed were prepared. The buffer solution was mixed with the rumen fluid in a 2:1 ratio while flushing the CO_2_ at 39 °C. About 30 mL buffer solution containing rumen fluid was dispensed in the individual syringe with the help of an automatic pipette (Varispenser, Eppendorf Vertrieb Deutschland GmbH, Wesseling-Berzdorf, Germany). The gas bubbles were gently pushed out of the syringe, and the initial piston position was recorded just prior to setting up the incubation. Generally, 24 h incubation is sufficient for the high protein feed ingredients, and as both the concentrate and OBW contained high CP, the 24 h incubation was sufficient for the fermentation. The samples were incubated in a *Hohenheim-type* water bath shaker at 39 °C for 24 h. The intermittent shaking was performed at every 4 h. The incubation was terminated the next day exactly after 24 h by placing the syringes in an ice tray, and the final position of the piston was recorded. The volume of total gas (mL) was calculated by the difference between the initial and final piston positions.

The gas sample from the glass syringe was quantitatively transferred to a pre-vacuumed serum glass vial (10 mL) fitted with a butyl stopper and aluminum crimp. For analyzing CH_4_ in a gas sample, about 1 mL of gas from the vials was drawn in an airtight glass syringe (1 mL, Hamilton Company, Reno, NV, USA) and 0.1 mL was presented to the gas chromatograph (7890B GC System, Agilent Technologies Inc., Santa Clara, CA, USA) equipped with a thermal conductivity detector and a porapak Q packed column (80/100, 6′, 2 mm ID). The gas chromatograph was operated with the following conditions: injector temperature 60 °C, column oven temperature 100 °C, and detector temperature 110 °C. The airflow rate was set at 400 mL per minute, while the flow rates of H_2_ and N_2_ were 40 and 30 mL per minute, respectively. Before analyzing actual samples, a CH4 standard of known concentration (21.8%) was injected three times in gas chromatography. The CH_4_ (%) was calculated using the following equation and finally expressed in mL per 200 mg of sample and mL per g of dry matter.

$${\mathrm{CH}}_{4}\;(\%)=\frac{\mathrm{sample}\;\mathrm{area}\;\times \;\mathrm{std}.\;\mathrm{concentration}}{\mathrm{std}.\;\mathrm{area}}$$

### 2.4. In Vitro Dry Matter Digestibility (IVDMD)

About 500 mg sample was placed in a 100 mL glass syringe (Haberle, RSA cutting technologies GmbH, Schwerte, Germany) and 40 mL buffered rumen inoculum was added as described in the previous section. The collection of rumen fluid, processing, weighing of samples, and incubation were the same as stated above. To determine the DMD, all the samples were incubated in sextuplicate (R = 6) for 24 h in a *Hohenheim-type* water bath shaker at 39 °C with intermittent shaking at every 4 h. The fermentation was terminated after 24 h by placing the syringes on ice, and the content through the syringe luer was transferred to a fiber bag (ST 100, C. Gerhardt GmbH & Co. KG, Königswinter Germany). The fiber bags were washed repeatedly until the water became clear, and thereafter, the bags were placed in a hot air oven for drying at 80 °C for 24 h. The IVDMD was determined by the difference in initial and final weight as given below.

$$IVDMD\;(\%)=\frac{intial\;sample\;weight\;(mg)-dried\;weight\left(mg\right)}{initial\;weight\;of\;sample}\times 100$$

### 2.5. Volatile Fatty Acid (VFA) and Ammonia-N

The spent incubation fluid obtained (R = 6) from the syringes on the termination of fermentation was centrifuged at 13,000× *g* at 4 °C for 15 min. The supernatant was transferred to another tube to estimate the amount of volatile fatty acids (VFA). The incubation fluid was mixed with 25% metaphosphoric acid (*v*/*v*) in a 4:1 ratio and stored at −80 °C until further analysis. The frozen samples were thawed at room temperature, followed by a brief centrifugation. About 0.5 mL of thawed sample was transferred to the autosampler vials (1.5 mL, Agilent) and placed into the autosampler of a gas chromatograph (7890B GC System, Agilent Technologies Inc., Santa Clara, CA, USA). The determination of the individual VFA concentrations was carried out following the method of Filípek and Dvořák [23] with minor modifications [24]. The concentration of VFA was determined using the following equation, and VFA concentration was expressed in millimoles (mM).

$$VFA\;con.\;(mM)=\frac{Peak\;area\;of\;sample\times Conc.\;of\;standard\times dilution}{Peak\;area\;of\;standard}$$

The ammonia-nitrogen in the incubation fluid was determined by following the standard procedure [25] and the titration was performed against 0.01 N sulfuric acid. Ammonia-N was calculated with the following equation:

$$Ammonia-N\;\left(mg/dL\;rumen\;fluid\right)=\;mL\;of\;0.01N\;H_{2}SO_{4}\times \;14$$

### 2.6. Protozoa Enumeration

Protozoa were enumerated (R = 6) by microscopic counting under a phase-contrast microscope (Eclipse Ci, Nikon Corporation, Tokyo, Japan) as per the method of Kamra and Agarwal [26]. The protozoa were morphologically categorized as *Entodiniomorphs* and *Holotrichs,* as described by Hungate [27]. 

### 2.7. DNA Extraction

Approximately 1.5 mL of incubation fluid (R = 6) containing both solid and liquid fractions was collected in a 2 mL Eppendorf tube. The DNA isolation was performed following the repeat bead beating and column (RBB + C) method as described by Yu and Morrison [28]. In brief, the samples were centrifuged at a speed of 12,000× *g* for 15 min. The supernatant was carefully removed, and 1 mL lysis buffer was added to dissolve the pellet. The dissolved pellet, along with the liquid fraction, was transferred to a pre-sterilized screw-cap tube (BioSpec Products, Bartlesville, OK, USA) that contained 0.5 g of zirconia beads (0.1 mm, BioSpec Products, Bartlesville, OK, USA). The samples were homogenized in a mini bead beater (BioSpec Products, Bartlesville, OK, USA) at the maximum speed for 3 min. Subsequently, the content was incubated at 70 °C for 15 min. Following centrifugation at 12,000× *g*, the supernatant was carefully collected in a 2 mL Eppendorf tube. In the screw-cap tube containing residue, 300 μL lysis buffer was added and performed the bead beating as described above and both the supernatant were then pooled. To precipitate the proteins and polysaccharides, the supernatant was treated with 260 μL of 10M ammonium acetate and placed on the ice for 5 min before centrifugation at 12,000× *g* at 4 °C for 10 min. Thereafter, the supernatant was removed in another tube, and an equal volume of isopropanol was added and mixed by the inversion of tubes. The DNA was collected by centrifugation at 4 °C for 10 min at 12,000× *g* and then the pellet was washed with 70% ethanol. The DNA pellet was dissolved in 100 μL Tris-EDTA buffer, and 2 μL DNase-free RNase (10 mg/mL, Qiagen GmbH, Hilden, Germany) was added to remove RNA contamination, followed by 15 min incubation at room temperature. After this step, the recommended QIAamp DNA mini kit (Qiagen GmbH, Hilden, Germany) was used for the isolation of DNA following the manufacturer’s instructions. The DNA quality was checked with 0.8% agarose gel electrophoresis and quantified by Qubit 4.0 (Invitrogen Corporation, Carlsbad, CA, USA).

### 2.8. Shotgun Metagenome Sequencing

The metagenomic DNA sequencing was performed on NovaSeq 6000 (Illumina Inc., San Diago, CA, USA) at Eurofins Genomics in Bangalore, India. The metagenomic libraries were prepared using NEBNext^®^ Ultra^TM^ II FS DNA Library Prep Kit (New England Biolabs, Ipswich, MA, USA). A quantity of DNA (100–500 ng) was fragmented to achieve a size of 350 base pairs. The fragmentation process was carried out using NEBNext Ultra II FS Reaction Buffer and Ultra II FS Enzyme Mix in a PCR thermal cycler. The fragmented DNA was ligated with the NEBNext Adaptor for Illumina through the process of combining 35 μL of fragmented DNA with the NEBNext Ultra II Ligation Master Mix. The mixture was then incubated at 20 °C for 15 min followed by ligation with adaptors. Thereafter, the PCR amplification was carried out using index primers (i5 and i7) under the following PCR conditions: initial denaturation at 98 °C, 30 s, denaturation at 98 °C, 10 s, annealing at 65 °C, 75 s, and final extension at 65 °C, 5 min. The PCR-enriched libraries were evaluated on Agilent 4150 Tape Station and then sequenced on NovaSeq 6000 to generate paired-end reads of 150 base pairs length.

### 2.9. Bioinformatics Analysis

Demultiplexed metagenomic raw reads were assessed for quality and adaptor contamination using FastQC v0.11.9 [29]. The leftover adapters, low-quality bases of Q < 30 and short reads (<100 bp), were filtered using trimmomatic v0.39 [30] with the following parameters: ILLUMINACLIP:TruSeq3-PE-2.fa:2:30:10 SLIDINGWINDOW:15:30 MINLEN:100 TRAILING:30 AVGQUAL:30. The quality filtered reads were mapped to cattle genome assembly ARS-UCD1.2 (RefSeq assembly accession: GCF_002263795.1) using BowTie2 v2.5.0 [31] to remove the host sequences and the unmapped reads were saved for further analysis. The unmapped reads from the previous step were taxonomically classified using the Kraken2 [32] and the full report output was sorted at different taxonomic levels in Pavian v1.2.0 [33]. The alpha and beta diversity of the metagenome was estimated at the genus level in MicrobiomeAnalyst v2.0 [34]. The alpha diversity was estimated by the Shannon index, whereas the beta diversity was assessed by the Bray-Curtis dissimilarity. The data was normalized using the total sum scaling (TSS) feature and analyzed at different taxonomic ranks in MicrobiomeAnalyst that converted the feature read counts clustered within the same taxonomic rank as a proportion of the total number of reads in the respective sample [35]. The metagenome data was analyzed using the Kruskal-Wallis test for the significance in ‘rstatix’ package v4.3.1, and the posthoc analysis (Dunn test) was performed to ascertain the significant difference between the treatments.

### 2.10. Gene Prediction and CAZyme Annotation

The paired and single-end clean reads free from host contamination were assembled in MEGAHIT v1.2.9 [36] with a minimum contig length cut-off at 500 bp. The prokaryotic genes in contigs were predicted by MetaGeneMark2 [37]. To remove the redundancy, the predicted gene sequences were clustered at a sequence identity threshold of 95% using CD-HIT-EST v4.8.1 [38]. The CAZyme families were identified in the non-redundant gene sequences using DIAMOND (v2.0.15.153), HMMER (v3.2.1), and dbCAN_sub databases in run_dbcan standalone version of the dbCAN3 [39]. The combined output files generated in DIAMOND, HMMER, and dbCAN_sub were filtered, and CAZymes predicted by at least two of the above tools were selected. Clean reads from each sample were mapped to the respective unigenes in the RNASeq analysis tool in CLC Genomics Workbench (v21.0.2; QIAGEN, USA). The gene abundance in each sample, expressed as transcripts per million (TPM), was added to the CAZy predictions. The CAZymes were categorized into different classes, namely auxiliary activities (AA), carbohydrate-binding modules (CBM), carbohydrate esterases (CE), glycoside hydrolases (GH), glycosyl transferases (GT), and polysaccharide lyases (PL). The abundance data was analyzed for statistical significance using Kruskal-Wallis, and the Dunn post hoc test was performed in R (version 4.3.1) to compare the mean values with significance between the treatments. The significant families were represented by heatmap using R-package ‘Pheatmap-1.0.12’ [40]. 

### 2.11. Statistical Analysis

The individual glass syringes representing the replicates (R = 6) for each treatment (*n*= 5) were considered experimental units, and the in vitro data were initially evaluated for the normal distribution (gaussian) using the built-in Shapiro-Wilk method in GraphPad Prism (v.9.0; GraphPad Software, San Diego, CA, USA). The data were analyzed in GraphPad Prism with one-way ANOVA using the following mathematical model:

$$Y_{ij}=µ+\tau_{i}+\epsilon_{ij}$$

where Y_ij_ represents the j^th^ observation (j = 1, 2, … 6) on the i^th^ treatment (i = 1, 2, … 5). µ was the common effect of the experiment, $\tau_{i}\;$represents the i^th^ treatment effect, and ∑_ij_ represents the random error due to the j^th^ observation of the i^th^ treatment

The mean values of the parameters with significant differences were compared using post hoc analysis in Tukey to determine the significance at a 95% confidence level. To ascertain the impact of inclusion levels of OBW in concentrate on total gas production, the simple linear regression was performed in GraphPad prism at 95% confidence intervals and the *p* value was calculated at 5% alpha threshold level. The Pearson correlation coefficient (r) between the levels of OBW, total gas and CH_4_ production was ascertained in GraphPad prism at an 0.05 alpha threshold. 

## 3. Results

### 3.1. Chemical Composition

**Figure 1:**
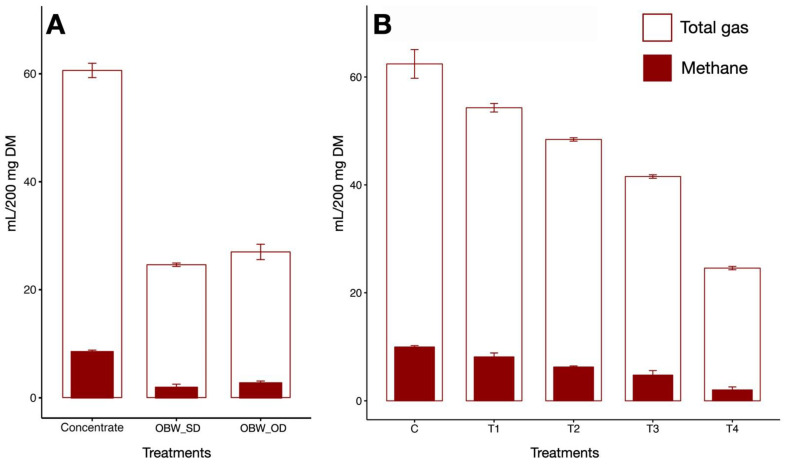
(**A**) Comparison of total gas (mL/200 mg DM) and CH_4_ (mL/200 mg DM) production between concentrate, OBW (shed; OBW_SD and open-air dry; OBW_OD) (**B**) effect of graded inclusion of OBW on total gas and CH_4_ production, the outer bars represent total gas, whereas inner dark bars represent CH_4_ production for the corresponding treatments. C (100 concentrate), T_1_ (80 concentrate—20 OBW), T_2_ (60 concentrate—40 OBW), T_3_ (40 concentrate—60 OBW), T_4_ (100% OBW).

The chemical composition analysis revealed that the OBW had a higher CP content as compared to concentrate (Table S1). The CP content of OBW was 9–10% higher than the concentrate. Likewise, the fiber content in OBW expressed as CF or fiber fractions, i.e., NDF and ADF, was considerably higher than the concentrate. The ash and organic matter content of the concentrate and OBW were similar. Results from Experiment I revealed that the OBW produced 67–77% less CH_4_ than the concentrate (Figure 1). However, CH_4_ production in OBW was not independent of the adverse action on feed fermentation as evidenced by the concurrent decrease in total gas production, about 55–59% lower in OBW.

### 3.2. CH_4_ Production

In vitro results demonstrated that the inclusion of OBW at the graded levels decreased CH_4_ production (mL/g DM) by 18, 37, 52, and 79% in T_1_, T_2_, T_3_, and T_4_ treatments, respectively (Table 1). The difference in CH_4_ production between C and T_1_ treatments was not significant, whereas the other three treatments (T_2_–T_4_) showed a significant decrease (*p* < 0.0001) in CH_4_ production as compared to C. The CH_4_ production between treatments T_2_ and T_3_ was not significantly different; however, the CH_4_ production in T_4_ was significantly different (*p* < 0.0001) from the treatments T_2_ and T_3_. The adjustment of CH_4_ production to a per-gram measurement of organic matter also revealed a similar trend, and the CH_4_ production in treatments T_2_, T_3_, and T_4_ was significantly (*p* < 0.0001) lower than the C. The reduction in CH_4_ production in treatments T_2_, T_3,_ and T_4_ as compared to C was 37, 52 and 80%, respectively. The results also established a substantial decrease of 27 to 38% in treatments T_2_ and T_3_ in CH_4_ production when the data was adjusted to dry matter digestibility in the corresponding treatments. Further, the CH_4_ production also showed a significant decrease in the correction of data to the organic matter digestibility (Table 1). Overall, a negative correlation was reported between OBW levels and CH_4_ production (r = −0.949) in the present study.

### 3.3. Total Gas

Results indicated a linear decrease (*p* < 0.0001) in total gas production (Table 1) with the graded incorporation of OBW from 20–60% (T_1_, T_2_, T_3_). Similarly, the total gas production with 20–60% inclusion of OBW in T_1_–T_3_ or with 100% OBW was significantly lower than the concentrate. The correlation between total gas production and levels of OBW in the concentrate was found to be negative (r = −0.986).

### 3.4. In Vitro Dry Matter Digestibility (IVDMD) and Organic Matter Digestibility (OMD)

The graded inclusion (T_1_–T_3_) of OBW at 20, 40, and 60% levels resulted in a substantial decrease (*p* < 0.0001) in DMD. Similarly, the IVDMD was decreased by 36% in treatment T_4_ as compared to treatment C (Table 1). Likewise, the graded inclusion of OBW also had a negative effect on the OMD. Data from the study established a negative correlation between the OBW inclusion levels and DMD (r = −0.977). 

### 3.5. VFA, Ammonia-N and Protozoa

Results from the study indicated that the VFA production was not adversely affected by the OBW inclusions in concentrate at the graded levels of 20–100% (Table 2). However, the valerate production was higher (*p* < 0.0001) in treatments T_3_ and T_4_, where OBW replaced 80 and 100% of the concentrate, respectively. The valerate production in all other treatments was similar. 

The results also indicated that replacing the concentrate with OBW at a rate of 60% or more (T_3_ and T_4_) resulted in a substantial increase of ammonia-N (mg/dL of rumen fluid) as compared to C (Table 2). The ammonia-N in T_1_ and T_2_ was similar to the concentration in treatment C. There was no effect of the concentrate replacement with OBW on the total protozoa (*p* = 0.604) and *Entodiniomorphs* (Table 2). Nevertheless, there was a noticeable decrease (*p* = 0.001) in the numbers of *Holotrichs* protozoa in the treatments T_3_ and T_4_. However, the numbers of *Holotrichs* in T_1_ and T_2_ were similar to that of the concentrate, i.e., control (C).

### 3.6. Alpha and Beta Diversity

**Figure 2:**
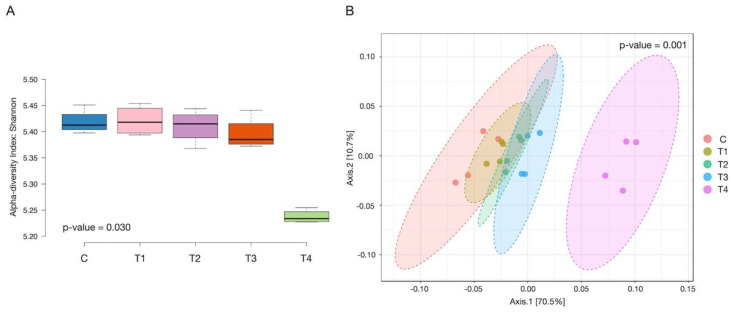
The figure displays alpha diversity using the Shannon index (**A**) and beta diversity using the Bray-Curtis index (**B**). C (100 concentrate), T_1_ (80 concentrate—20 OBW), T_2_ (60 concentrate—40 OBW), T_3_ (40 concentrate—60 OBW), T_4_ (100% OBW).

The alpha and beta diversity of the microbial communities are presented in Figure 2. The alpha diversity, denoted by the Shannon index, indicated a significant difference (*p* = 0.030) in the microbial genera richness among the treatments. The microbial genera richness in treatment T_4_ was considerably less than the other treatments, viz: C, T_1_, T_2_, and T_3_ (Supplementary File S1). However, the genera richness among the other treatments was similar and did not show any significant difference. The beta diversity represented by the Bray-Curtis diversity index indicated that there were significant (*p* = 0.001) differences in the microbial communities. The species community differences between C and T_3_, C and T_4_, T_1_ and T_4_, T_2_ and T_4_, and T_3_ and T_4_ treatments were significant (Supplementary File S1).

### 3.7. Metagenome Composition

**Figure 3:**
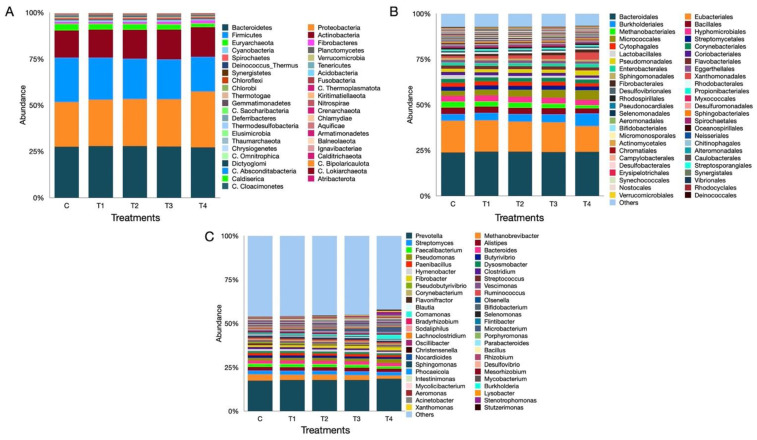
The figure shows the effect of graded replacement of concentrate with OBW on the microbial diversity at the phylum (**A**), order (**B**), and genus level (**C**). C (100 concentrate), T_1_ (80 concentrate—20 OBW), T_2_ (60 concentrate—40 OBW), T_3_ (40 concentrate—60 OBW), and T_4_ (100% OBW).

The rumen metagenome sequencing, with an average of 11.26 million reads per sample, generated a total of 225 million reads (Supplementary File S1). A total of 3.88 million reads were removed during the quality filtration in Trimmomatic. About 0.03% of reads per sample had host contamination, which was also removed before the downstream processing of data. A total of 43 phyla, 200 orders, and 1721 microbial genera were identified in this study (Supplementary File S1). The Bacteroidetes, Proteobacteria, Firmicutes, Actinobacteria, and Euryarchaeota were dominant microbial phyla in our study. These phyla represented more than 90% of the overall rumen microbial community (Figure 3A). The abundance of Bacteroidetes, one of the top f phyla, was similar across all the treatments (*p* = 0.489) and was not affected by the inclusion of OBW in the concentrate. Conversely, the abundance of Firmicutes decreased drastically (*p* = 0.007) as the inclusion level of OBW in the concentrate mixture increased. 

The disparity in the prevalence of Firmicutes between the C and T_4_ treatments was statistically significant. In a similar way, the distribution of the Euryarchaeota was negatively impacted (*p* = 0.009) by the OBW inclusion, and there was an apparent difference between the C and T_4_ treatments. In contrast, a significant increase (*p* = 0.016) in the abundance of Proteobacteria was observed with the higher levels of OBW in the concentrate. The differences in the abundance of Proteobacteria between the C and T_4_ treatments were significant (*p* = 0.010). The F/B ratio was adversely affected by the graded supplementation of OBW, which in turn affected the overall fermentation of the diet. The effect of the F/B ratio on the fermentation was also proved in our study by the decreased IVDMD and OMD (Table 1). 

**Figure 4:**
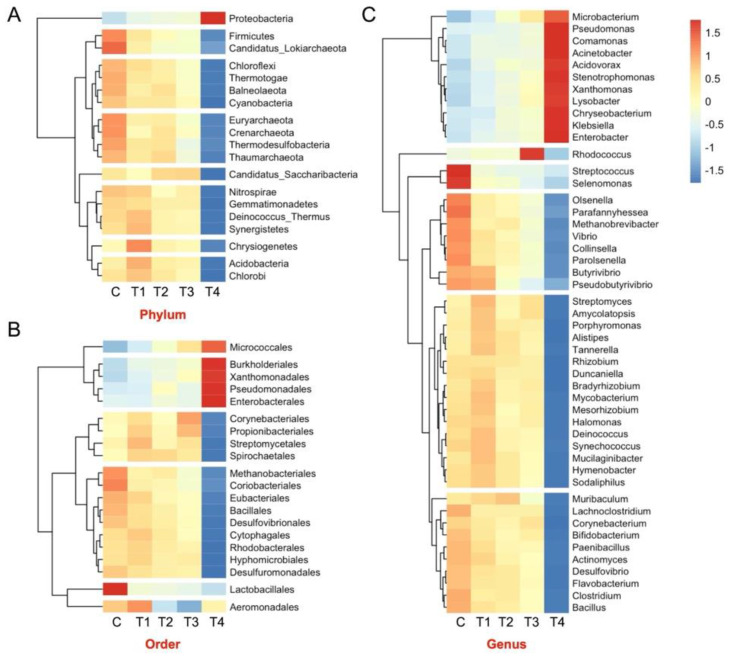
Heatmap representing the clustering of significantly different microbes at the phylum (**A**), order (**B**), and genus level (**C**). C (100 concentrate), T_1_ (80 concentrate—20 OBW), T_2_ (60 concentrate—40 OBW), T_3_ (40 concentrate—60 OBW), and T_4_ (100% OBW).

The heatmap derived from the data indicated that 19 distinctive microbial phyla were grouped into 8 distinct clusters. The Proteobacteria, Candidatus_Saccharibacteria, and Chrysiogenetes were each grouped into a single cluster. The Firmicutes and Candidatus_Lokiarchaeota, as well as the Acidobacteria and Chlorobi, constituted two distinct clusters. In contrast, there were three clusters, each including four phyla (Figure 4A).

The Bacteroidales were the most abundant order, making up approximately one-fourth of the whole ruminal microbiota (Figure 3B). Nevertheless, there was no significant difference (*p* = 0.77) in the distribution among the treatments. The Eubacteriales was the second largest order, and the distribution of microbes belonging to this order exhibited a significant difference in the distribution (*p* = 0.03) among the treatments. The abundance of Eubacteriales decreased in a linear fashion as the supplementation level of OBW increased in the concentrate. The differences between the C and T_4_ treatments were significant. Within the archaeal domain, the Methanobacteriales displayed the most dominant order (Figure 3B), ranked as the fifth largest order in the ruminal microbiota in the present study.

The findings revealed that the abundance of Microbacteriales was significantly reduced (*p* = 0.009) due to the graded inclusion of OBW in the concentrate. The difference between the C and T_4_ treatments was significant, but the Methanobacteriales among the other treatments were similar. In an identical manner, the abundance of Halobacteriales archaea was also negatively impacted (*p* = 0.044) by the supplementation of OBW. The Methanomassiliicoccales ranked third in abundance among the archaea (Supplementary File S1); nonetheless, their distribution was similar across the treatments. Out of the top 20 significantly different orders, the microbiota was clustered into six groups. The largest cluster, i.e., the Methanobacteriales, consisted of 9 orders in the present study (Figure 4B). Likewise, the cluster consisting of Bukholderiales and Corynebacteriales contains four orders within each cluster. Conversely, Micrococcales, Lactobacillales, and Aeromonadales are each represented by a single order in a cluster. 

At the genus level, the *Prevotella* constituted the largest fraction of the ruminal microbiota (~20%). However, their distribution was similar (*p* = 0.44) among the groups. The second most abundant genus was *Methanobrevibacter* (Figure 3C)*,* which constituted 2–2.5% of the microbiome and was also significantly different (*p* = 0.009) between the C and T_4_ groups (Figure 3C). Similarly, the abundance of *Methanosphaera* (*p* = 0.03) and *Methanobacterium* (*p* = 0.004) between the C and T_4_ groups was significantly different. On the other hand, the distribution of *Methanomassiliicoccus* and *Methanosarcina* was similar among the groups. The microbiota results revealed a similar distribution of *Bacteroidetes*, a fibrolytic genus, among the group (*p* = 0.363). However, the distribution of *Butyrivibrio* was significantly different among groups (*p* = 0.045). Another important genus accountable for fibrolytic activity, i.e., *Ruminococcus,* was also similar among groups. *Streptococcus*, another fibrolytic genus, was adversely affected by the OBW supplementation, and there was a significant difference (*p* = 0.006) between the C and T_4_ groups. The top 50 most abundant and significantly different microbes were grouped into six clusters, with the *Streptomyces* cluster being the largest, comprising 16 genera. Similarly, the *Muribaculum* cluster also comprised 10 genera (Figure 4C). On the other hand, the *Steptococcus* and *Rhodococcus* were the smallest clusters, comprising two members and one member, respectively.

### 3.8. CAZyme Profiles

**Figure 5:**
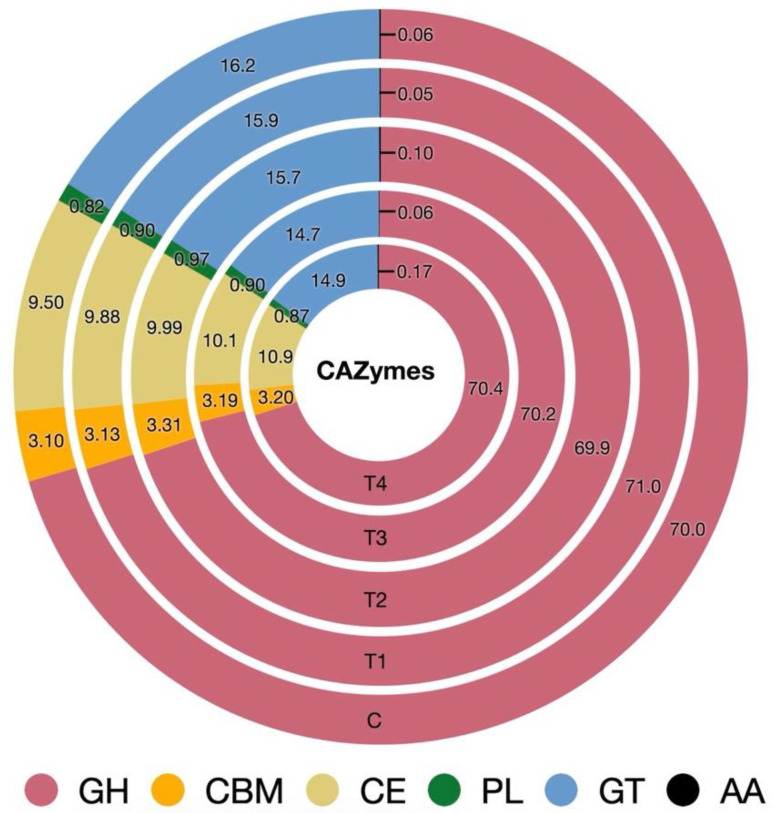
Effect of graded replacement of concentrate with OBW on the distribution of CAZymes classes. C (100 concentrate), T_1_ (80 concentrate—20 OBW), T_2_ (60 concentrate—40 OBW), T_3_ (40 concentrate—60 OBW), and T_4_ (100% OBW). GH—glycoside hydrolases, CBM—carbohydrate-binding modules, CE—carbohydrate esterases, PL—polysaccharide lyases, GT—glycosyl transferases, AA—auxiliary activities.

The processed raw data assembled into contigs and used for gene prediction yielded 3.78 million unique genes. The mean number of unigenes was 0.14, 0.20, 0.22, 0.20, and 0.19 million in C, T_1_, T_2_, T_3_, and T_4_, respectively. The unigenes were used to predict the CAZymes, resulting in 2.59–3.1% of the unigenes being assigned to CAZymes. In the present study, a total of six CAZymes classes, namely auxiliary activities (AA), carbohydrate-binding modules (CBM), carbohydrate esterases (CE), glycoside hydrolases (GH), glycosyl transferases (GT), and polysaccharide lyases (PL) were identified. Among the classes, the GH constituted the largest fraction of CAZymes (~70%), whilst the GT was found to be the second most abundant class. The minimum abundance was assigned to the AA CAZymes class. Results from the study indicated a significant difference in the abundances of CAZymes affiliated with the CBM, CE, GH, and GT classes (Figure 5, Supplementary File S1). The abundance of CBM and CE CAZymes significantly increased (*p* = 0.015) in treatment T_4_. On the contrary, the abundances of GT CAZymes decreased (*p* = 0.012) due to the complete replacement of concentrate with OBW (T_4_). The analysis of the CAZymes profile revealed that out of the total 233 CAZymes families, six belonged to class AA, 35 to CBM, 16 to CE, 111 to GH, 45 to GT, and 20 to PL.

**Figure 6:**
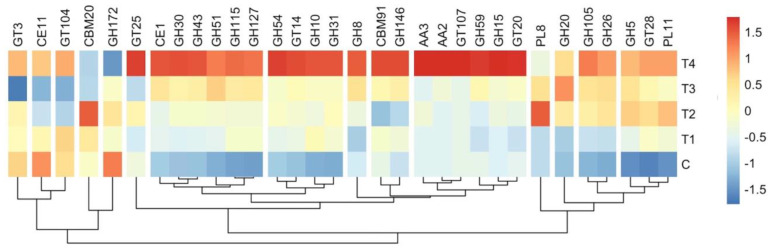
A heatmap representing the clustering of significantly different CAZyme among the treatments. AA—auxiliary activities, CBM—carbohydrate-binding modules, CE—carbohydrate esterases, GH—glycoside hydrolases, GT—glycosyl transferases, and PL—polysaccharide lyases. C (100 concentrate), T_1_ (80 concentrate—20 OBW), T_2_ (60 concentrate—40 OBW), T_3_ (40 concentrate—60 OBW), T_4_ (100% OBW).

The replacement of concentrate with OBW at graded levels impacted a total of 30 CAZymes families (Supplementary File S1). The CAZymes of 30 GH families were affected by the OBW inclusion, and their abundances increased with the incremental levels. A significant difference in abundance was observed between the C and T_4_ treatments. A total of 8 CAZymes that belonged to the GT families were affected by the OBW inclusion, and the abundances of most of these CAZymes were significantly increased. The heatmap displayed the grouping of distinct CAZyme families into four major and 11 minor groups (Figure 6). The two largest clusters each contained six families, and the one that possessed the function of hemicellulose degradation revealed that CE1, GH43, and GH51 families were most abundant and significantly different between the treatments. 

## 4. Discussion

Approximately 200 g of brewing waste is generated for every liter of beer produced, resulting in a yearly waste generation of 37–40 million tons [14,41,42]. About 85% of the waste produced by the breweries consists of solid materials and is primarily disposed of in landfills [43]. A limited fraction of the brewery waste is utilized for livestock feeding. Given its current price of 30–35 euro (~3000 INR) per ton [44], brewery waste is significantly cheaper than the prevailing price of concentrate in India, which is approximately 30,000 INR per ton. The comparison based on nutrient density revealed that OBD had 1.5 times greater CP density than the concentrate, whereas the energy density was marginally higher. Consequently, brewery waste could serve as a cost-effective and excellent alternative to partially replace the expensive concentrate in livestock feeding. The present study demonstrated that OBW had higher CP, fiber, and ether extract content than the concentrate mixture. These findings are consistent with previous reports [15,41,45]. The increment in the fiber content with the graded replacement of concentrate with OBW in this study could be due to the removal of starch and other soluble sugars during the malting and messing process [15]. The higher protein content in OBW than the concentrate can be attributed to the protein-rich malt generally used in the brewing process [46]. In this study, we have reported a comparatively higher ether extract content in OBW than in the concentrate. Westendorf and Wolt [15] reported a higher EE content in the brewer waste than their foundation grains. The EE content in OBW in this study was in good agreement with Kaur and Saxena [47]. On the contrary, few studies have reported low EE content in brewers’ waste [46,48], primarily due to the long duration used for the fermentation.

The reduction of CH_4_ production can be attributed to a number of mechanisms, including alterations to the composition of the diet [49,50], direct inhibition of methanogens [51,52], decrease in the protozoa population [53,54,55], alterations to the metabolic pathways that are linked to the supply of substrate, oil supplementation [56] and secondary metabolites in plants [57,58]. In this study, the graded replacement of concentrate with OBW (20–100%) resulted in a reduction of 18–79% in CH_4_ production. The low dry and organic matter digestibility in the test treatments (T_1_–T_4_) can partially explain the decrease in CH_4_ production. The disproportionate decrease in digestibility and reduction in CH_4_ production of the treatments indicated that the lower digestibility was not only the factor accountable for the whole reduction, but there were other mechanisms by which the graded inclusion of OBW affected CH_4_ production, too. These results corroborate previous reports that the reduction in digestibility is one of the major factors for less CH_4_ production [59,60].

Results from the study indicated a gradual decrease in dry matter and organic matter digestibility on the incorporation of OBW at the graded levels in concentrate. Our findings established that the total gas, dry matter, and organic matter digestibility was negatively correlated with the inclusion of the level of OBW, which cautioned the inclusion of the OBW at an appropriate level. The decrease in digestibility can be attributed to the higher crude fiber, NDF, and ADF contents of the OBW as compared to the concentrate. The crude fiber content was almost 5 times greater in OBW than that of concentrate (Table S1). The reduction in digestibility with increasing levels of OBW was in agreement with previous studies [46,61,62], which may be attributed to the digestion of non-fiber carbohydrates (NFC), mainly starch, during the malting process [63]. The remaining NFC is likely to be resistant to microbial degradation [64]. A greater VFA transport has been reported from the ruminal lumen to the epithelium in animals fed on moist reconstituted grains rather than dry [65]. The genes regulating the VFA transport through proteins may have a synergistic association with VFA concentration [66]. Despite the reduction in digestibility, the similar VFA production except valerate indicated that the partial replacement of concentrate with OBW did not lead to a shift in the fermentation pattern. Like our findings, Benedeti et al.’s [65] study on cattle reported a greater concentration of valerate on the feeding of reconstituted grains. This study showed that the ruminal ammonia-N concentration increased when the concentrate mixture was gradually replaced with OBW. The amount of ammonia-N produced by proteolysis and subsequently used for microbial protein synthesis determines the concentration of ammonia-N in the rumen [67]. Furthermore, it has been reported that a diet high in starch has a lower ammonia-N concentration due to the significant influence of non-fiber carbohydrates [68]. Thus, the higher fiber fractions (CF, NDF, and ADF) in the test treatments relative to the control can account for the higher ammonia-N.

In the present study, the *Entodiniomorphs* remain unaffected by the supplementation of OBW, whereas the *Holotrichs* were substantially decreased with the 80 (T_3_) and 100% (T_4_) replacement of concentrate with OBW in treatments T_3_ and T_4_. Approximately 9–25% of the rumen methanogens are associated with protozoa [69], and therefore, any alterations in their numbers can lead to a change in the CH_4_ production [70] via interspecies H_2_ transfer from protozoa to methanogens [71]. Holotrichs are reported to have greater CH_4_ production ability than *Entodiniomorphs* without impacting the fermentation or digestibility [72]. Despite the smaller numbers of *Holotrichs* in the rumen, the elimination led to a decrease in CH_4_ production due to their strong association with the most active methanogens community, i.e., PAM [73].

The prevalence of Bacteroidetes, Firmicutes, and Proteobacteria in this study aligns with previous research [74,75,76,77]. Perhaps due to its role in fermenting complex carbohydrates and its abundance of CAZyme-encoding genes, the inclusion of OBW did not affect the abundance of Bacteroidetes. The presence of Firmicutes is typically linked to the energy density of the diet. The decline in Firmicutes levels with higher levels of OBW may be due to the reduced digestibility of dry matter and organic matter despite the higher ether extract content in OBW treatments compared to the control. The results align with those of Liu et al. [78], who reported that a high-grain diet resulted in a notable increase in Firmicutes. The concentrate used in the control treatment consisted of 32% maize grain, which gradually decreased by 6.4% with each increasing level of OBW in treatments T_1_, T_2_, and T_3_. The prevalence of Proteobacteria in the rumen microbiome aligns well with earlier research [79,80]. *Proteobacteria* abundance decreases as dietary fiber levels increase, but high protein content leads to their increased abundance [81]. In our study, the increased abundance of *Proteobacteria* may be linked to the high protein content in the test treatments. 

The high abundance of Euryarchaeota within the archaeal phyla corresponds with the previous studies [56,77,82,83]. Thaumarchaeota and Crenarchaeota, belonging to the superphyla TACK, were identified in the present study [84]. Members of the superphyla DPANN were absent from the findings of our study. Euryarchaeota, due to the inclusion of all methanogens in this group, was the fifth-largest phylum and the most prominent one in the archaeal community. Our findings indicated that OBW inclusion has an adverse impact on the abundance of Euryarchaeota. The OBW adversely affected Methanobacteriales, a prominent order of Euryarchaeota. Our findings indicated that the abundance of Euryarchaeota is adversely impacted by the inclusion of OBW. The dominance of Methanobrevibacter in the archaeal community is in consonance with the previous reports [82,85].

Methanogenesis is most feasible through the hydrogenotrophic pathway [86], and *Methanobrevibacter* is a prominent hydrogenotrophic methanogen in the rumen [87,88]. Therefore, less substrate (H_2_) availability to hydrogenotrophic methanogens due to the lower numbers of Holotrichs and depression in digestibility may lead to a significant reduction in CH_4_ production in OBW-incorporated treatments. These findings are consistent with Wang et al. [89], who reported that higher production of H_2_ yields more CH_4_. We have not performed the microbial identification up to the species level; nevertheless, great variability in CH_4_ production has been reported among the *Methanobrevibacter*. The abundance of the SGMT clade was found to be positively correlated with CH_4_ production [90,91,92], whereas the RO clade produces relatively less CH_4_.

The metagenome data at the order level and phenotypic data on CH_4_ production (mL/200 mg) revealed that the abundances of Methanobacteriales (r = 0.949) and Methanomassiliicoccales (r = 0.955) were found to be positively correlated with CH_4_ production. At the genus level, *Methanobrevibacter*, *Methanomassiliicoccus*, *Methanosphaera*, *Methanosarcina*, *Methanococcus*, and *Methanocaldococcus* were found to be positively correlated (r = 0.93) with CH_4_ production. These findings indicated that the reduction in CH_4_ production coincided with the decreasing abundances of the above archaeal genera (Supplementary File S1). 

OBW also had an adverse impact on the abundance of *Methanosphaera*, a methanol-utilizing methanogen. A study by Pitta et al. [93] supported our findings, confirming that *Methanosphaera* is positively correlated with CH_4_ production, and that is why their abundance decreased with the incremental incorporation of OBW. The similar distribution of Methanomassiliicoccales, a group of methylotrophic methanogens, indicated that their abundance was not influenced by the OBW incorporation. Similarly, the similar abundance of *Methanosarcina* in different treatments could be due to their high H_2_ threshold, and probably the usual concentration of H_2_ in the rumen does not support their growth [94].

CAZymes of microbial origin are accountable for driving the carbohydrate fermentation in the rumen. CAZymes classes were identified in this study, and the overall dominance of GH and GT classes is in good agreement with our previous study in cattle and buffaloes [83,95]. GH is a broad class consisting of a large number of families (111 identified in this study) and is involved in the degradation of cellulose, chitin, and starch [96,97]. CAZymes affiliated with the GH hydrolyze the glycosidic bonds between a sugar and non-sugar moiety [98], while GT leads to the hydrolysis of glycosidic bonds in proteins, nucleic acids, and oligosaccharides [99]. Recently, it has been reported that the abundance of the GH3 family was relatively higher in high-forage-fed animals than in low-forage-fed animals [100]. These findings support our results for the overall dominance and higher abundances of the GH3 family with the incremental change in OBW levels.

In India, about 85% of the livestock, including cattle, buffalo, sheep, and goats, are reared by small and marginal farmers and are accountable for more than 95% of CH_4_ emissions [101]. Marginal and landless farmers have meager money to spend on the purchase of concentrate to sustain production and CH_4_ mitigation. The partial replacement of concentrate with OBW anywhere in the world will not only economize the cost of feed formulation but will also curtail the large contribution of livestock to GHG emissions. However, a concurrent decrease in fermentation cautioned for the inclusion of OBW at an appropriate level at which the fermentation parameters remain uncompromised. 

## 5. Conclusions

From the study, it can be inferred that the partial replacement of concentrate with OBW significantly decreases CH_4_ production by reducing the Holotrichs protozoa and altering rumen microbiota. However, despite a substantial decrease in CH_4_ production at the highest level, a concurrent depression in digestibility cautions its inclusion at a safe level where fermentation characteristics remain unaffected. OBW could be a cheaper alternative for the concentrate mixture to minimize the contribution of livestock to CH_4_ emissions. To confirm the efficacy of CH_4_ reduction and the potential impact of OBW on productive traits such as milk and meat production, animal studies with the variable proportions of OBW in roughage-concentrate diets need to be conducted.

## Figures and Tables

**Table 1:** Effect of OBW inclusion levels on total gas, CH_4_, and IVDMD.

Attributes	Treatment					SEM	*p*
	C	T_1_	T_2_	T_3_	T_4_		
Total gas (mL/g DM)	312 ^a^	271 ^b^	242 ^c^	208 ^d^	123 ^e^	11.05	<0.0001
CH_4_ (mL/g DM)	49.8 ^a^	40.8 ^a^	31.4 ^b^	23.9 ^b^	10.1 ^c^	4.92	<0.0001
CH_4_ (mL/g OM)	52.7 ^a^	43.0 ^a^	32.9 ^b^	25.0 ^b^	10.5 ^c^	7.29	<0.0001
IVDMD (%)	83.7 ^a^	79.3 ^a^	72.9 ^b^	64.7 ^c^	47.3 ^d^	2.56	<0.0001
OMD (%)	75.3 ^a^	71.6 ^b^	66.4 ^c^	58.5 ^d^	41.6 ^e^	0.855	<0.0001
Total gas (mL/g dig. DM)	373 ^a^	343 ^b^	333 ^b^	321 ^b^	260 ^c^	15.28	<0.0001
CH_4_ (mL/g dig. DM)	59.5 ^a^	51.5 ^ab^	43.1 ^b^	36.8 ^b^	21.2 ^c^	7.83	<0.0001
CH_4_ (mL/g dig. OM)	70.1 ^a^	60.1 ^ab^	49.6 ^bc^	42.6 ^c^	25.2 ^d^	7.67	<0.0001

CH_4_—methane, DM—dry matter, dig. DM—digestible dry matter, OM—organic matter, OMD—organic matter digestibility, IVDMD—in vitro dry matter digestibility, SEM—standard error of the mean, C (100 concentrate), T_1_ (80 concentrate—20 OBW), T_2_ (60 concentrate—40 OBW), T_3_ (40 concentrate—60 OBW), T_4_ (100% OBW). *p*—level of significance at <0.05. Mean values bearing different superscripts in a row differ significantly.

**Table 2:** Effect of graded replacement of concentrate by OBW on VFA (mM), ammonia-N (mg/dL rumen fluid), and protozoa population.

Attributes	Treatment					SEM	*p*
	C	T_1_	T_2_	T_3_	T_4_		
**VFA**							
Acetate	67.6	67.9	66.4	67.9	67.1	2.01	0.829
Propionate	23.2	23.1	20.5	22.1	18.9	3.84	0.502
Butyrate	4.57	5.49	6.46	5.04	6.08	1.00	0.104
Valerate	2.07 ^a^	2.29 ^ab^	2.44 ^ab^	3.02 ^c^	3.29 ^c^	0.18	<0.0001
Iso-valerate	2.67	1.67	2.43	0.18	0.49	1.47	0.102
A/P ratio	2.93	2.95	3.33	3.31	3.55	0.62	0.565
Ammonia-N	16.1 ^a^	18.9 ^ab^	19.2 ^ab^	21.0 ^b^	22.4 ^b^	1.06	0.005
**Protozoa**							
Total (×10^7^ cells/mL fluid)	8.27	8.24	8.21	8.07	8.01	0.050	0.604
Entodiniomorphs (×10^7^ cells/mL fluid)	8.25	8.22	8.19	8.06	8.00	0.048	0.647
Holotrichs (×10^6^ cells/mL fluid)	0.213 ^a^	0.214 ^a^	0.213 ^a^	0.153 ^b^	0.137 ^b^	0.016	0.001

SEM—standard error of the mean, VFA—volatile fatty acids, A/P—acetate to propionate ratio. C (100 concentrate), T_1_ (80 concentrate—20 OBW), T_2_ (60 concentrate—40 OBW), T_3_ (40 concentrate—60 OBW), T_4_ (100% OBW). *p*—level of significance at <0.05. Mean values bearing different superscripts in a row differ significantly.

## Data Availability

The datasets presented in this study can be found in online repositories with the accession number PRJNA1018446. The metagenome data with accession number(s) is available in the repository/repositories and can be found at: https://www.ncbi.nlm.nih.gov/sra/PRJNA1018446 (accessed on 19 September 2023).

## Supplementary Materials

The following supporting information can be downloaded at https://www.mdpi.com/article/10.3390/microorganisms12071475/s1, Table S1: Chemical Composition of oat brewery waste. Supplementary File S1: Metagenome read statistics, phylum and genus level abundance data in and CAZyme profile of experimental groups.
