# Supplementary material for: Oat Brewery Waste Decreased Methane Production and Alters Rumen Fermentation, Microbiota Composition, and CAZymes Profiles
Source: Microorganisms. 2024 Jul 19;12(7):1475. doi: 10.3390/microorganisms12071475 (PMC11279122; doi:10.3390/microorganisms12071475)
Supplement: Supplementary file 1 [file microorganisms-12-01475-s001.zip › Table S1.pdf]

Table S1: Chemical Composition of oat brewery waste

**Treatment**		Composition (% DM basis)					
	**OM**	**CP**	**CF**	**NDF**	**ADF**	**EE**	**Ash**
Control (100* conc. – 0 OBW)	94.2	20.0	4.58	22.8	6.75	1.53	5.77
T_1 (80 conc. - 20 OBW)	94.7	22.0	8.03	28.5	10.4	1.66	5.33
T_2 (60 conc. - 40 OBW)	95.3	25.5	9.79	39.0	15.0	2.28	4.70
T_3 (40 conc. - 60 OBW)	95.8	25.8	12.9	50.5	19.5	2.46	4.21
T_4 (0 conc. – 100 OBW)	96.6	29.8	20.1	76.1	27.4	2.75	3.42
**Oat brewery waste**							
OBW-SD	96.6	29.8	20.1	76.1	27.4	2.75	3.42
OBW-OD	96.5	30.1	21.0	77.9	28.6	2.73	3.51

DM- dry matter, C- control (100% concentrate – 0% OBW), OBW- oat brewery waste, OM- organic matter, CP- crude protein (N x 6.25), CF- crude fibre, NDF- neutral detergent fibre, ADF- acid detergent fibre, EE- ether extract, SD- shed dry, OD- oven dry. The mean values of the chemical constituents are based on three observations (R = 3). * figures in parenthesis is proportion (percentage) of concentrate and OBW.
